# Points of view in understanding trilobite eyes

**DOI:** 10.1038/s41467-021-22227-8

**Published:** 2021-04-07

**Authors:** Brigitte Schoenemann, Euan N. K. Clarkson

**Affiliations:** 1 Department of Zoology, Neurobiology/Animal Physiology and Biology Education, University of Cologne, Cologne, Germany; 2 Grant Institute, School of Geosciences, The Kings Buildings, University of Edinburgh, Edinburgh, UK

**Keywords:** Palaeontology

**Arising from** Scholtz et al. *Nature Communications* 10.1038/s41467-019-10459-8 (2019).

The internal structure of compound eyes offers important insights into the phylogeny of their bearers. Crystalline cones are present in mandibulate arthropods, such as crustaceans and insects^1,2^. The older subphylum Chelicerata, including horseshoe crabs and arachnids, does not have crystalline cones^3,4^. The earliest Palaeozoic trilobites putatively had crystalline cones^5^. Many trilobites that lived after the Cambrian apparently did not. Here we argue that a recent attempt to show that an Ordovician asaphid trilobite possessed crystalline cones^6^ failed because the structures considered are the relics of elongated prismatic lenses, and consequently likely are artefacts. There is one individual structure, however, suggesting the possible presence of a system comparable to crustacean compound eyes, with a flat lens and an elongated crystalline cone, probably highly refractive. We suggest that trilobites may have had two visual systems, one system with powerful calcitic lenses without a crystalline cone (functionally a chelicerate type: e.g., some Asaphids, Phacopids), or having just a reduced crystalline cone^7^ and another system equipped with a flat ineffective lens and an elongated refractive crystalline cone (crustacean type: e.g., *Archegonus*).

Trilobites are extinct arthropods that dominated the Palaeozoic. They were equipped with elaborate compound eyes from their very beginning ~521 mya (million years ago). Phylogenetically, they have been considered to be related to chelicerates (e.g., spiders, scorpions, horseshoe crabs)^8^, but other authorities see them as more closely related to mandibulates (crustaceans, hexapods and myriapods)^9^. The internal structure of compound eye units (ommatidia) in chelicerates and mandibulates differs, amongst other points, in that the Mandibulata possess a cellular crystalline cone below each lens. This character, which could inform the phylogenetic placement of trilobites, has recently been suggested to have been present in trilobites^5,7^. Scholtz and co-workers took up this point^6^, and claimed the existence of crystalline cones in two different species of trilobites. Here we illustrate that their first example of a putative crystalline cone, where the main emphasis was laid, is just a weathering artefact. The situation of the second example also remains vague due to the indistinct quality of preservation. We succeeded, however, in finding in the same specimen a structure, suggesting a similarity to the crystalline cones of the Jurassic crustacean *Dollocaris ingens* van Straelen 1923^10^. Both systems, the one of the Jurassic crustacean as of the trilobite here (*Archaegonus*) are charcterised by a thin, more or flat lens, which due to the missing curved refracting surfaces probably had low or no focusing power. Consequently, one may infer that the found structure underneath this lens was a crystalline cone, that took over this function. We suggest that this part of the specimen may be investigated more closely.

Firstly, however, to assess the relevance of this discussion about the existence of crystalline cones and their significance to trilobite phylogeny, it is necessary to understand how a trilobite compound eye, especially its dioptric apparatus, is constructed. Schoenemann et al.^5^ showed that the most common type of trilobite compound eye, the holochroal eye, in principle is a so-called apposition eye, still common in many extant diurnal arthropods, and that this system is more than half a billion years old^5^. A compound eye typically consists of up to several tens, hundreds, or sometimes even thousands of identical units, the ommatidia. In terrestrial arthropods, each has a cuticular lens, focusing light through a cellular crystalline cone onto a light guiding structure (rhabdom). The rhabdom is part of the receptor cells, transforming the light energy into an electrical signal. The receptor cells are linked to the central nervous system, where the signal is processed and interpreted. In total, the image seen is mosaic-like^3,11^. In aquatic systems, however, the difference of the refractive indices between water and the organic material of the lens is not high enough to establish efficient refractive power, so aquatic arthropod eyes normally have no effective cuticular lenses. This function is taken over by an adapted crystalline cone which works as an index gradient lens or alternatively by other mechanisms. Thus the dioptric apparatus of an aquatic apposition eye (Mandibulata) typically consists of a thin, ineffective cuticular lens, and a powerful refracting crystalline cone. The only group of arthropods with a very different system are trilobites. Here the mineral lenses are of pure calcite with a high refractive index^12,13^. In consequence, the focal lengths are very short, and, especially in those systems with more or less spherical lenses, effectively no crystalline cone was needed. Chelicerate compound eyes possess no crystalline cones^3,4^, those of the horseshoe crab, *Limulus*, and probably of the extinct eurypterids focus the light through index gradient exocones formed by the cuticle^3,4,14^. The idea that the lenses of trilobites were formed primarily of calcite was challenged recently^15^, but evidence for this challenge is currently lacking.

The first trilobite where evidence for an ommatidium could be established is *Schmidtiellus reetae* Bergström, 1973 (~520 mya) from the Cambrian Series 2, Stage 3 (Lower Cambrian) of Estonia^5^ (Fig. 1a, f). This trilobite displays the typical apposition eye concept–consisting of (relics of) receptor cells arranged like rosettes, a central rhabdom, and putatively a crystalline cone (Fig. 1b–e, j). All these structures are united to a columnar ommatidium^5^. There are some unusual features of this eye, especially that the ommatidia are situated in wide cellular ‘baskets’ different from those of modern eyes.

**Fig. 1:**
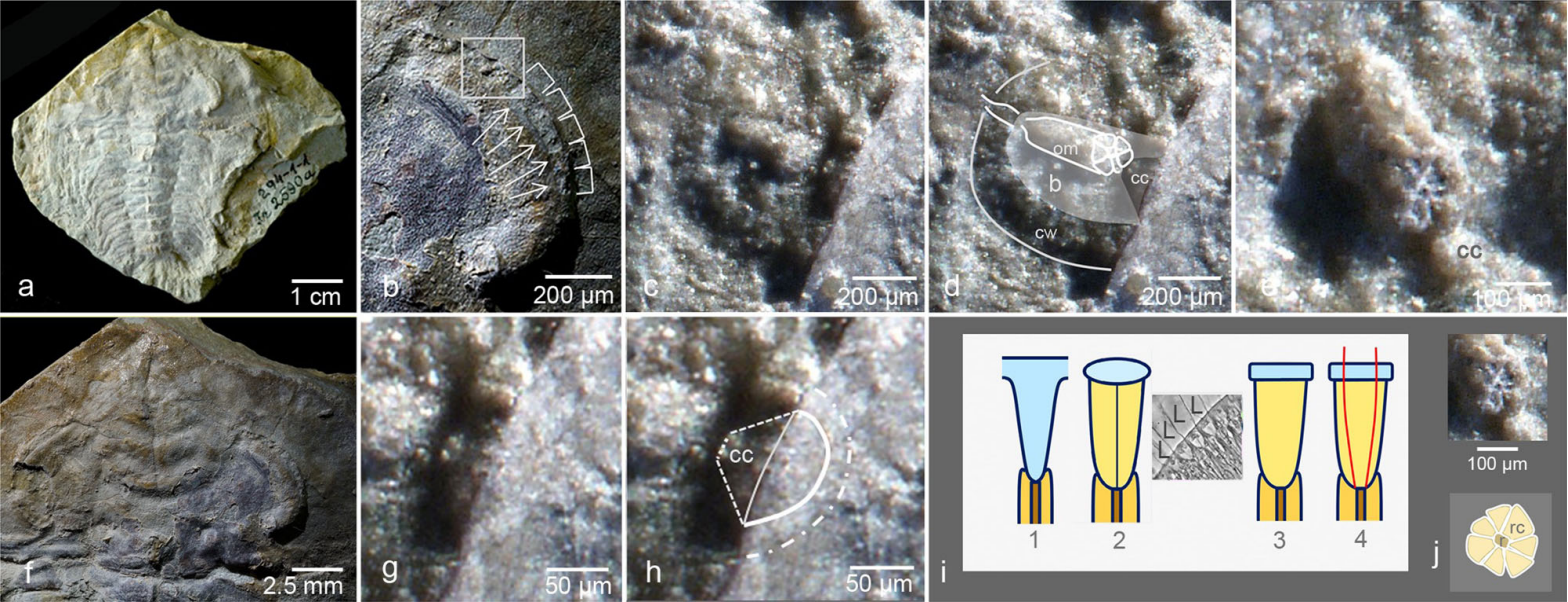
Apposition compound eye and its fossil record. **a** Trilobite *Schmidtiellus reetae* Bergström, 1973, (deposited at the Institute of Geology at Tallinn University of Technology, Estonia, under repository number GIT 294-1., holotype), Lower Cambrian, Estonia, and its compound eye. **b** Abraded part of the right eye. Note the rectangular sequential elements ‘baskets’ containing the ommatidia. **c**, **d** ‘Basket’ with ommatidium. **e** Ommatidium in cross section, showing clearly relics of receptor cells, a central rhabdom and crsytalline cone. **f** head of *S. reetae* (a). **g**, **h** Top part of **c**. Crystalline cone and thin cuticle above the ommatidia. **i** Principles of optics in arthropods. 1 Exocone of Xiphosura (Chelicaerata, *Limulus*), 2 insects, insert: ommatidia of the honey bee (*Apis mellifera* L.) with a thick, effective cuticular lens in a histological section. Note the crystalline cones below the lenses., 3, 4 crustacean, with ray path in an index gradient lens. **j** cross-section and schematic drawing of the ommatidium in *S. reetae*. b ‘basket’, cc crystalline cone, ce cellular wall of the ‘basket’, om ommatidium, L lens, r rhabdom, rc receptor cell; green: lens, pink: crystalline cone, yellow: receptor cell, brown: rhabdom.

Scholtz et al.^7^ describe fossilised substructures of a rhabdom, microvilli containing the visual pigments. This, however, seems hardly possible as the size of these substructures is commonly known to lie between ~40 and 120 nm^16^, p. 384 which is too small to be distinguished from grains of the petrified fossil.

The lenses of (holochroal) trilobite eyes often consist of small, elliptical lenses, covered by a thin membrane, the cornea. Trilobites with thick shells, however, are equipped with elongated ‘prisms’, as is elaborately described by Lindström^17^, which later was confirmed and physically analysed by Clarkson^18,19^. Typical representatives of such thick-shelled trilobites with lens-prisms are trilobites of the genus *Asaphus* (*Asaphidae, Asaphida*) (Fig. 2h, m–p, u–x). The lenses of all trilobites known are constructed of radial lamellae, thickening towards the edges during growth^18^. In the centre the lamellae are thinner, they lie closer to each other and thus achieve the highest density. This weak centre of the lenses dissolves more readily than does the periphery, a process that results in cavities embraced by a steep ring of the rest-material, which appears in section as a sharp spine^17^ (Fig. 2b, c, d, q, s).

**Fig. 2:**
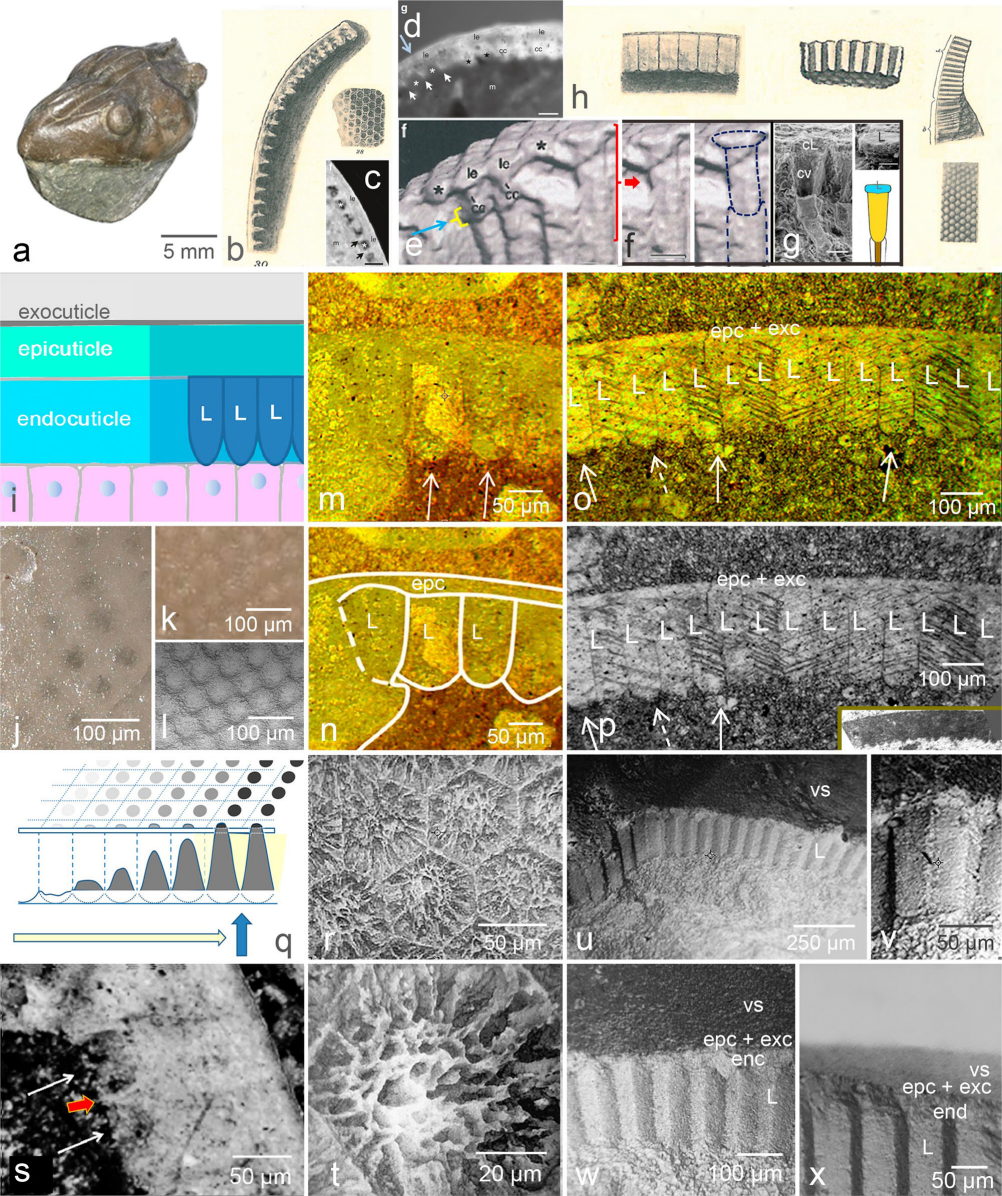
The optical system of asaphid trilobites. **a** *Asaphus* (*Neoasaphus*) *expansus* (Wahlenberg, 1821), Orthoceras limestone, Ordovician, Sweden [GIK 201]. **b** Lindström´s drawing (1901) of lenses hollowed out by decay, continuously weathered from the periphery inwards. Second drawing: View on top of the relics of the lenses, which are filled with dark mud^17^. **c**, **d** [=Fig. 2f, g^6^] The same specimen, black arrows from Fig. 2f^6^ indicate spine-shaped relics of the weathered prismatic lenses, mistaken in^6^ as crystalline cones (d, bright blue arrow added to Fig. 2f^6^ indicates dividing line between two former lenses). **e** [=Fig. 3f^6^] *Archegonus wahrsteiniensis*, blue arrow and yellow curly bracket, added to the original figure, indicate atypical structure; red curly bracket, added to the original figure Fig. 3f^6^ indicates a section, shown in **f**. **f** putative thin lens and crystalline cone, (indicated in blue broken lines, added to the original figure), similar to those of **g** *Dollocaris ingens*^10^. **h** Cylindrical elongated lenses (‘prisms'^17^) of *A. expansus* in the endocuticle, not to be mistaken for crystalline cones, drawn by Lindström^17^. **i** Schematic drawing of the structure of the cuticle of arthropods (darkened those parts which can be seen in fossils). **j** Visual surface of *Nileus* sp. [GIK 202], showing seen from top the phenomenon of mud-filled prismatic lenses, hollowed out by weathering and filled with dark mud. **k**, **I** Intact visual surface of *A. raniceps* [GIK 203]. **m**, **n** Parabolic to hemicircular refractive inner surfaces of these prismatic lenses [GI R 5011]. **o** Sequence of prismatic lenses [GI R 5011]. **p** o in black and white to enhance the contrast, making the boundaries between the lenses more visible [GI R 5011]. **q** Model to illustrate, how ‘spines’ and hollow-spaces develop during proceeding weathering [GI R 5011]. **r**, **t** Initial hollowing out of the lenses seen from the top (*Paladin eichwaldi shunnerensis* (King 1914)), [GR I 45668]. **s** Initial hollowing out of the lens-prisms (note the arising spine between the arrows) [GI R 5011]. **u** Evidence of elongated lenses in intact visual surfaces of *A. raniceps* [GIR 5505], as described by Lindström^17^. Note the elongated lenses below the exocuticle. **v** Individual lens. **w** Section of the visual surface, showing the exocuticle (dark layer), epicuticle and endocuticular lens prisms. cL cavity of the lens, cv cavity of the crystalline cone, enc endocuticle, epc epicuticle, exc exocuticle, L lens, vs visual surface. [**c** synchrotron; **e**, **f** µ-ct; **o**–**r** thin-sections under polarised light; **d**, **j**, **k**, **r** light microscope, **g**, **I**, **s**–**x** SEM. scales: **c** 20 µm, **d** 40 µm, **e** 50 µm, **g** 10 µm.

Considering the material of Scholtz et al.^6^ (Fig. 2) closely, we do not observe the elongated lens-prisms. The reason is explained by Lindström, though missing in the discussion of Scholtz et al.^6^ using the same specimen: In a view from the top ″… they [the lenses] have the shape of hollow, white rings filled with black mud, and in a longitudinal section the white walls of the lenses look like short pointed spikes and interiorly they are completely empty.″^17^ p. 42 (See Fig. 2c, d [=Fig. 2f,g in ref. ^6^], showing the relevant part of the Lindström specimen (Fig. 2b) as a synchrotron scan. Figure 2q–t indicates that dissolution of the lower parts of the prismatic lenses by spreading diagenesis results in the spikes, which Scholtz et al.^6^ interpret as crystalline cones. The semi-thin section (Fig. 2d [=Fig. 2g^6^]) clearly shows a boundary between two lenses as a dark contour [bright blue arrow added to the original figure Fig. 2g in ref. ^6^, here Fig. 2d]). Thus, the characteristic elongated prismatic lenses are missing from the fossil, and the structures that were interpreted as crystalline cones are more probably artefacts: the marginal remains of the dissolved lens-prisms.

Scholtz et al.^6^ tried to support their idea of existing crystalline cones based on the partially dissolved prismatic lenses of *Asaphus* by microphotographs, µ-ct and SEM of the eye of another trilobite *Archegonus wahrsteiniensis*, which is hardly convincing. The photomicrographs do not provide any differentiation between a lens and any crystalline cone, the µ-ct (Fig. 2e [=Fig. 3f in ref. ^6^]) shows just one example similar to a crystalline cone in a random surrounding (so the crystalline cones may be a random-structure too); consequently any persuasive evidence of the existence of crystalline cones is missing here. Furthermore, there is a rather atypical structure directly below the triangular element which was interpreted as a crystalline cone, generating doubts to the interpretation that these structures are genuine (Fig. 2e, [=Fig. 3f in ref. ^6^], blue arrow, and yellow curly bracket indicating the atypical structure were added to the original Fig. 3f in ref. ^6^). Finally, in the enlarged and accentuated illustration of Fig. 3e^6^, no clear differentiation between the elements of the dioptric apparatus can be distinguished in the randomly distributed material of the fossil, and those possibly contained are covered by suggestive outlines. There is just one singular element that may represent a crystalline cone, but it was not described in Scholtz et al.^6^. It can be seen at the right side of the µ-ct (Fig. 2e [=Fig. 3f in ref. ^6^]. A section of Fig. 2e, f [=Fig. 3f in ref. ^6^] marked by the added red curly bracket in Fig. 2f [=Fig. 3f in ref. ^6^] shows clearly that the element looks almost identical to the elongated crystalline cones, situated below a flat, thin lens found in the Jurassic crustacean *Dollocaris ingens*^10^ (Fig. 2g). In an aquatic system this concept of a less refractive lens and a powerful focusing crystalline cone seems to be repeated here in the trilobite *A. wahrsteiniensis*.

We conclude that the *Asaphus* crystalline cones described by Scholtz et al.^6^ are artefacts, resulting from partial dissolution. The triangular spikes, previously interpreted as crystalline cones, more probably represent the relics of the dissolved elongated prismatic lenses, and the supporting material does not provide convincing evidence of differentiated elements. Thus, the crystalline cones suggested by Schoenemann et al.^5,7^ are the only robust indications of trilobite crystalline cones that we have so far. One may speculate that because of the high refractive capacity of calcitic lenses of trilobites, the phylogenetically implicated, but functionally dispensable, crystalline cones often were reduced or even lost after the earliest Cambrian forms. We found a vague indication in the material of *Archegonus wahrsteiniensis*, not discovered by the authors^6^, which coincides with the crystalline cones found in the Jurassic crustacean *Dollocaris ingens*^10^. It may be worthwhile to reconsider this specimen of *A. wahrsteiniensis* to determine the existence of crystalline cones in trilobites.

Our new findings of a likely existing elongated crystalline cone underneath the flat lens of *A. wahrsteiniensis* suggest that there may have been two systems in trilobites: (1) one manifesting the mandibulate type with a thin, flat, less functional lens, and an elongated, probably highly refracting crystalline cones, such as that of *Dollocaris*^10^ and many modern crustaceans (e.g., *Archegonus*), and (2) another with elongated or spherical highly refracting calcitic lenses (e.g., genus *Asaphus*, Phacopids) without or with reduced crystalline cones^3,7^, functionally comparable to the cheliceratean eyes of the xiphosuran *Limulus* and probably eurypterids. However, any discussion of the implications of the crystalline cone for the assignment of trilobites to the Mandibulata instead of to the Chelicerata may become obsolete if crystalline cones are ever found in a genuine stem arthropod. There is good evidence that compound eyes existed in this early stem-line or stem-line-close arthropods^20^.

## Methods

The specimens illustrated here are: *Schmidtiellus reetae Bergström, 1973*, Lükati Fm., Atdabanian, Lower Cambrian, Saviranna, Estonia, stored in the collection of the Institute of Geology at Tallinn University of Technology, Estonia, GIT 294-1 [Fig. 1]; *Asaphus* (*Neoasaphus*) *expansus* (Wahlenberg, 1821), Orthoceras limestone, Ordovician, Sweden, Geologisches Institut der Universität zu Köln, Germany, GIK 201 [Fig. 2a, k, l]; *Asaphus* sp., Lindström specimen, Ordovician, Gotska sandön, Gotland, Sweden, Naturhistoriska Riksmuseet, Sektionen för Paleozoologi, Stockholm, Sweden, Ar0059402 [Fig. 2c, d]^17^; *Archegonus* (*Waribole*) *warsteinensis* (Rud. & E. Richter, 1926), Fammenian, Upper Devonian, Kalvarienberg/Kallenhardt, Germany, Museum für Naturkunde Berlin, Germany, MB.T 7303, [Fig. 2e, f]^6^; *Dollocaris ingens* Van Straelen, 1923, Early Callovian, La Voulte-sur-Rhône Lagerstätte, Middle Jurassic, Ardèche, France, Muséum National d’Histoire Naturelle, Paris, France, MNHN.F.A29278, [Fig. 2g]^10^. *Nileus* armadillo (Dalman, 1827), Kunda Fm., Lower Ordovizium, Östergötland/ Schweden, Geologisches Institut der Universität zu Köln, Germany, GIK 202, [Fg. 2j]; *Asaphus raniceps* Dalman, 1827, Llanvirnian, Lower Ordovician, Haget, northern Oeland, Sweden, Grant Institute of Geology, University of Edinburgh, Scotland, GI R 5011, [Fig. 2m–p, s]; *Paladin eichwaldi shunnerensis* (King 1914), Namurian, Mid Carboniferous, Shunner Fell Well, Great Shunner Fell, West Yorkshire, England. GI R 45668 [Fig. 2r, t]; *Asaphus ranicep*s Dalman, 1827, lower Llanvirnian, Ordovician, Haget, Öland, Sweden, Grant Institute of Geology, University of Edinburgh, Scotland, GI R 5505, [Fig. 2u–x].

The specimens here newly documented were photographed with a Keyence digital microscope (VHX-700F, objective VH-Z20T).

### Reporting summary

Further information on research design is available in the Nature Research Reporting Summary linked to this article.

## Supplementary information

Reporting Summary

## Data Availability

The authors confirm that all relevant material is available as noted under Methods.
