# Supplementary material for: Points of view in understanding trilobite eyes
Source: Nat Commun. 2021 Apr 7;12:2081. doi: 10.1038/s41467-021-22227-8 (PMC8027602; doi:10.1038/s41467-021-22227-8)
Supplement: Supplementary file 1 — Reporting Summary [file 41467_2021_22227_MOESM1_ESM.pdf]

nature research

Corresponding author(s): Brigitte Schoenemann

Last updated by author(s): 2020 09 14

Reporting Summary

Nature Research wishes to improve the reproducibility of the work that we publish. This form provides structure for consistency and transparency in reporting. For further information on Nature Research policies, see our Editorial Policies and the Editorial Policy Checklist.

Statistics

For all statistical analyses, confirm that the following items are present in the figure legend, table legend, main text, or Methods section.

n/a	Confirmed	
☒	☐	The exact sample size (*n*) for each experimental group/condition, given as a discrete number and unit of measurement
☒	☐	A statement on whether measurements were taken from distinct samples or whether the same sample was measured repeatedly
☒	☐	The statistical test(s) used AND whether they are one- or two-sided<br>*Only common tests should be described solely by name; describe more complex techniques in the Methods section.*
☒	☐	A description of all covariates tested
☒	☐	A description of any assumptions or corrections, such as tests of normality and adjustment for multiple comparisons
☒	☐	A full description of the statistical parameters including central tendency (e.g. means) or other basic estimates (e.g. regression coefficient) AND variation (e.g. standard deviation) or associated estimates of uncertainty (e.g. confidence intervals)
☒	☐	For null hypothesis testing, the test statistic (e.g. F, t, r) with confidence intervals, effect sizes, degrees of freedom and P value noted<br>*Give P values as exact values whenever suitable.*
☒	☐	For Bayesian analysis, information on the choice of priors and Markov chain Monte Carlo settings
☒	☐	For hierarchical and complex designs, identification of the appropriate level for tests and full reporting of outcomes
☒	☐	Estimates of effect sizes (e.g. Cohen's *d*, Pearson's *r*), indicating how they were calculated

Our web collection on statistics for biologists contains articles on many of the points above.

Software and code

Policy information about availability of computer code

Data collection	No software was used for data collection.
Data analysis	No software was used for data analyses, figures were prepared with and considered under Photoshop Elements 11.

For manuscripts utilizing custom algorithms or software that are central to the research but not yet described in published literature, software must be made available to editors and reviewers. We strongly encourage code deposition in a community repository (e.g. GitHub). See the Nature Research guidelines for submitting code & software for further information.

Data

Policy information about availability of data

All manuscripts must include a data availability statement. This statement should provide the following information, where applicable:

- Accession codes, unique identifiers, or web links for publicly available datasets
- A list of figures that have associated raw data
- A description of any restrictions on data availability

The palaeontological specimens are deposited in a registered museum or collection to permit free access by other rearchers in perpetuity.

Field-specific reporting

Ecological, evolutionary & environmental sciences study design

All studies must disclose on these points even when the disclosure is negative.

Study description	With thin-sections of the Grant Institute, Edinburgh we could show that the evidence of essential structures of mandibulate compound eyes, the crystalline cones, as had been postlated by Scholtz and coworkers in 2019 for trilobites, was insufficient, because the main structure they found was a preservational artefact, and the second structure they presented was not convincing. We suggested, however, to reconsider that specimen used here, (Archegonus warsteinensis), because at one place in the specimen figured, different from the ones described by Scholtz et al.(2019), we found a structure very similar to crystalline cones in the Jurassic crustacean Dllocaris ingens (Vannier et al. 2016).
Research sample	Thin-sections of trilobite eyes. Asaphus raniceps, A. expansus., Paladin eichwaldi shunnerensis, are all deposited in the Grant Institute, School of Geosciences, University of Edinburgh. Nileus sp. is deposited in the collection of the Geological Institute of the University of Cologne.
Sampling strategy	The thin-sections were investigated microscopically, with a KEYENCE digital light microscope, VH-X 700F.
Data collection	The investigation refers to museum specimens.
Timing and spatial scale	n/a
Data exclusions	no data exclusions
Reproducibility	n/a
Randomization	n/a
Blinding	n/a

Did the study involve field work? ☐ Yes ☒ No

Reporting for specific materials, systems and methods

We require information from authors about some types of materials, experimental systems and methods used in many studies. Here, indicate whether each material, system or method listed is relevant to your study. If you are not sure if a list item applies to your research, read the appropriate section before selecting a response.

Materials & experimental systems

n/a	Involved in the study	
☒	☐	Antibodies
☒	☐	Eukaryotic cell lines
☐	☒	Palaeontology and archaeology
☐	☒	Animals and other organisms
☒	☐	Human research participants
☒	☐	Clinical data
☒	☐	Dual use research of concern

Methods

n/a	Involved in the study	
☒	☐	ChIP-seq
☒	☐	Flow cytometry
☒	☐	MRI-based neuroimaging

Palaeontology and Archaeology

Specimen provenance	Schmidtiellus reetae Bergström, 1973, Lükati Fm., Lower Cambrian, Saviranna, Estonia,[Fig. 1]; Asaphus (Neoasaphus) expansus (Wahlenberg, 1821), Orthoceras limestone, Ordovician, Sweden [Fig. 2a,k,l]; Asaphus sp., Lindström specimen, Ordovician, Gotska sandön, Gotland, Sweden, [Fig 2c,d,]4; Archegonus (Waribole) warsteinensis (Rud. & E. Richter, 1926), Fammenian, Upper Devonian, Kalvarienberg/Kallenhardt, Germany, [Fig. 2 e,f]4; Dollocaris ingens Van Straelen, 1923, Early Callovian, La Voulte-sur-Rhône Lagerstätte, Middle Jurassic, Ardèche, France,MNHN.F.A29278, [Fig. 2g]14. Nileus armadillo (Dalman, 1827), Kunda Fm., Lower Ordovizium, Östergötland/ Schweden, GIK 202, [Fg. 2j]; Asaphus raniceps Dalman, 1827, Llanvirnian, Lower Ordovician, Haget, northern Oeland, Sweden [Fig. 2m-p,s]; Paladin eichwaldi shunnerensis (King 1914), Namurian, Mid Carboniferous, Shunner Fell Well, Great Shunner Fell, West Yorkshire, England [Fig. 2r,t]; Asaphus raniceps Dalman, 1827, lower Llanvirnian, Ordovician, Haget, Öland, Sweden, [Fig. 2 u-x]. The specimens Scholtz at al. (2019) reported about: the specimens of Asaphus sp. were collected by Gustav Lindström, end of the 19th century, and published 1901; the specimen of Archegonus warsteinensis was collected by Dieter Korn, at Kalvarienberg, Kallenhardt, Germany.
Specimen deposition	Schmidtiellus reetae Bergström, 1973, Lükati Fm., Lower Cambrian, Saviranna, Estonia, stored in the collection of the Institute of Geology at Tallinn University of Technology, Estonia, GIT 294-1 [Fig. 1]; Asaphus (Neoasaphus) expansus (Wahlenberg, 1821), Geologisches Institut der Universität zu Köln, Germany, GIK 201 [Fig. 2a,k,l]; Asaphus sp., Lindström specimen, Naturhistoriska Riksmuseet, Sektionen för Paleozoologi, Stockholm, Sweden, Ar0059402 [Fig 2c,d,]4; Archegonus (Waribole) warsteinensis (Rud. & E. Richter, 1926), Museum für Naturkunde Berlin, Germany, MB.T 7303, [Fig. 2 e,f]4; Dollocaris ingens Van Straelen, 1923, Muséum National d'Histoire Naturelle, Paris, France, MNHN.F.A29278, [Fig. 2g]14. Nileus armadillo (Dalman, 1827), Geologisches Institut Universität zu Köln, GIK 202, [Fg. 2j]; Asaphus raniceps Dalman, 1827, Grant Institute of Geology, University of Edinburgh, Scotland, GI R 5011, [Fig. 2m-p,s]; Paladin eichwaldi shunnerensis (King 1914), Grant Institute of Geology, University of Edinburgh, Scotland, GI I R 45668 [Fig. 2r,t]; Asaphus raniceps Dalman, 1827, Grant Institute of Geology, University of Edinburgh, Scotland, GI R 5505, [Fig. 2 u-x]. Lindström´s specimens of Asaphus sp. are deposited at the Naturhistorika Riksmuseet, Sektionen för Palaeozoologi, Stockhlom, Sweden; the specimen of Archegonus warsteinesis is deposited in the Musem für Nturkunde, Berlin, Germany.
Dating methods	The specimens here newly documented were photographed with a Keyence digital microscope (VHX-700F, objective VH-Z20T)

☒ Tick this box to confirm that the raw and calibrated dates are available in the paper or in Supplementary Information.

Ethics oversight	No ethical approval or guidance is required, because our trilobites are petrified.

Note that full information on the approval of the study protocol must also be provided in the manuscript.

Animals and other organisms

Policy information about studies involving animals; ARRIVE guidelines recommended for reporting animal research

Laboratory animals	No laboratory animals
Wild animals	n/a
Field-collected samples	n/a
Ethics oversight	-

Note that full information on the approval of the study protocol must also be provided in the manuscript.
